# Renal Atrophy Relates to Brain Volume Loss on 7,149 MRI Scans

**DOI:** 10.1002/alz.094081

**Published:** 2025-01-09

**Authors:** Somayeh Meysami, Cyrus A. Raji, Sam Hashemi, Saurabh Garg, Nasrin Akbari, Ahmed Gouda, Yosef Gavriel Chodakiewitz, Thanh Duc Nguyen, Kellyann Niotis, David A. Merrill, Rajpaul Attariwala

**Affiliations:** ^1^ Pacific Brain Health Center, Pacific Neuroscience Institute and Foundation, Santa Monica, CA USA; ^2^ Mallinckrodt Institute of Radiology, Washington University, St. Louis, MO USA; ^3^ Prenuvo, Vancouver, BC Canada; ^4^ Vigilance Health Imaging Network Inc., Vancouver, BC Canada; ^5^ The Institute for Neurodegenerative Diseases (IND) Florida, Boca Raton, FL USA

## Abstract

**Background:**

Renal atrophy may reflect an end organ consequence of chronic vascular disease. Renal volume loss may therefore provide a window into brain aging and Alzheimer disease risk.

**Method:**

We obtained whole‐body 1.5T MRI scans on 7,149 healthy individuals across four sites. The participants had a mean age of 53.06 ± 12.95 years (age range: 18‐97 years), with a gender distribution of 48% women and 52% men; 38% were non‐white. Deep learning with FastSurfer on 3D T1 volumetric MPRAGE trained on 134 participants aged 27‐66 and segmented 96 brain regions. Whole body sequences included coronal T1 that allowed for separate segmentation and quantification of renal volumes. Partial correlation analysis controlling for sex and total intracranial volume was done on kidney volumes and the following brain regions: tissue types, lobar structures and Alzheimer disease risk regions – hippocampus, precuneus, and posterior cingulate gyrus. Renal volumes were normalized to the aggregate abdominal organ volumes and tissues such as the liver, spleen and abdominal fat. Multiple comparisons were corrected for with the Bonferroni method.

**Result:**

Lower renal volumes were correlated with lower gray matter volumes (rp = ‐0.229, p = 5.18×10‐85) as well as total white matter volume (rp = ‐0.204, p = 2.51×10‐67). The cerebral ventricles, demonstrated a moderate positive correlation with lower renal volumes (rp = 0.099, p = 5.28×10‐16) reflective of a generalized parenchymal volume loss. Lower renal volumes also predicted lower lobar volumes in: frontal lobe (rp = ‐0.204, p = 3.34×10‐67), temporal lobe (rp = ‐0.236, p = 9.76×10‐90), parietal lobe (rp = ‐0.215, p = 2.69×10‐74), and occipital lobe (rp = ‐0.207, p = 8.29×10‐69), all showed significant negative correlations with lower renal volumes. Lower renal volumes also correlated to lower volumes in AD risk regions: the Hippocampus (rp = ‐0.203, p = 2.89×10‐66) and posterior cingulate (rp = ‐0.18, p = 3.06×10‐52), precuneus (rp = ‐0.171, p = 8.00×10‐47).

**Conclusion:**

This study demonstrates a strong relationship in a large sample between lower renal volumes and reduced volumes in various brain regions, including gray matter, white matter, and specific lobar structures, as well as Alzheimer's disease risk regions.

